# Treatment of Primary Autoimmune Cerebellar Ataxia with Mycophenolate

**DOI:** 10.1007/s12311-020-01152-4

**Published:** 2020-06-10

**Authors:** M. Hadjivassiliou, R. A. Grunewald, P. D. Shanmugarajah, P. G. Sarrigiannis, P. Zis, V. Skarlatou, N. Hoggard

**Affiliations:** 1 Academic Department of Neurosciences, Sheffield Teaching Hospitals NHS Trust, Sheffield, UK; 2 Department of Neurology, Evaggelismos General Hospital, Athens, Greece; 3 IICD insigneo, University of Sheffield, Sheffield, UK

**Keywords:** Immune ataxias, Primarry autoimmune cerebellar ataxia, Mycophenolate, MR spectroscopy

## Abstract

Immune-mediated ataxias account for a substantial number of sporadic otherwise idiopathic ataxias. Despite some well-characterised entities such as paraneoplastic cerebellar degeneration where diagnostic markers exist, the majority of immune ataxias remained undiagnosed and untreated. We present here our experience in the treatment of suspected primary autoimmune cerebellar ataxia (PACA) using mycophenolate. All patients reported attend the Sheffield Ataxia Centre on a regular basis and had undergone extensive investigations, including genetic testing using next-generation sequencing, with other causes of ataxia excluded. The diagnosis of PACA was strongly suspected based on investigations, pattern of disease progression, and cerebellar involvement. Patients were treated with mycophenolate and monitored using MR spectroscopy of the cerebellar vermis. Thirty patients with PACA are reported here. Of these, 22 received mycophenolate (group 1). The remaining 8 were not on treatment (group 2-control group). Out of the 22 treated patients, 4 underwent serial MR spectroscopy prior to starting treatment and thus were used as controls making the total number of patients in the control group 12. The mean change of the MRS within the vermis (NAA/Cr area ratio) in the treatment group was + 0.144 ± 0.09 (improved) and in the untreated group − 0.155 ± 0.06 (deteriorated). The difference was significant. We also demonstrated a strong correlation between the spectroscopy and the SARA score. We have demonstrated the effectiveness of mycophenolate in the treatment of PACA. The results suggest that immune-mediated ataxias are potentially treatable, and that there is a need for early diagnosis to prevent permanent neurological deficit. The recently published diagnostic criteria for PACA would hopefully aid the diagnosis and treatment of this entity.

## Introduction

Immune-mediated ataxias include paraneoplastic cerebellar degeneration (PCD), gluten ataxia (GA) and post-infective cerebellitis. Unlike PCD, GA and post-infective cerebellitis where an antigenic trigger is known, in most suspected autoimmune ataxias, the antigenic trigger is not known and any associated neuronal antibodies are not well characterized or proven pathogenic. The term Primary Autoimmune Cerebellar Ataxia (PACA) was introduced to describe this later group [1].

Progress in our ability to genetically characterise the ataxias, using next-generation sequencing has, paradoxically, also resulted in appreciating that the majority of idiopathic sporadic ataxias are not due to a genetic defect. Adult-onset idiopathic sporadic ataxias account for 20% of all ataxias [2].

We suspect that a substantial number of such patients have an immune-mediated ataxia along the lines of PACA. This suspicion is not just based on the absence of any genetic defect but also the acute/subacute onset of ataxia (unlike genetic ataxias), the predilection for vermian involvement, the concurrent presence of other autoimmune diseases and additional clues from other investigations (e.g. CSF pleocytosis, OCB’s, presence of neuronal and non-neuronal antibodies). Criteria aiding the diagnosis of PACA have recently been published [3]. Here, we report our experience in treating patients suspected of having PACA using mycophenolate.

## Material and Methods

### Patient Selection

All patients reported here attend the Sheffield Ataxia Centre on a regular basis. These patients have undergone extensive investigations for an underlying cause of their ataxia, including genetic testing using NGS and other tests. Full details of such investigations have been published previously [2]. In all patients reported here, other causes of ataxia were excluded and the diagnosis of PACA was suspected based on the acute/subacute onset of ataxia, the predilection for vermian involvement both clinically and on imaging, the concurrent presence of other autoimmune diseases and additional clues from other investigations (e.g. CSF pleocytosis, OCB’s, presence of neuronal and non-neuronal antibodies) [3]. This information is summarised in Table 1.

**Table 1:** Clinical characteristics of 30 patients with PACA. Cerebellar atrophy was rated as 1 = mild, 2 = moderate, 3 = severe with a mean score for vermian atrophy 1.44 vs hemispheric atrophy of 1. This demonstrates the preferential involvement of the vermis in PACA. *IDDM* insulin dependent diabetes mellitus, *PA* pernicious anaemia, *SPS* stiff person syndrome, *SLE* systemic lupus erythematosus

Average age at onset of ataxia	56 years (range 18 to 83)
Average age at the time of this report	63 years (range 20 to 86)
Average duration of ataxia	7 years (range 2 to 20)
additional autoimmune diseases	100% (10 thyroid disease, 5 IDDM, 4 PA, 3 Sjogren’s, 2 SPS, 1 SLE, 1 scleroderma, 1 RA, 1 Crohn’s, 1 myositis, 1 vitiligo)
CSF oligoclonal bands	5/10 (50%)
auto-antibodies	26/30 (87%) (12 thyroid peroxidase, 5 low level anti-GAD, 4 intrinsic factor, 3 anti-Ro, 1 ANA/dsDNA, 1 centromere, 1 anti-CCP)
Subacute presentation	21/30 (70%)
Acute presentation	4/30 (13%)
Gait ataxia	100%
Abnormal vermian NAA/Cr	100% (mean NAA/Cr ratio 0.82)
Degree of vermian atrophy	1.44 (1 mild, 2 moderate, 3 severe)
Degree of hemispheric atrophy	1

It is our current practice to treat patients with suspected PACA who appear to follow a progressive course with mycophenolate. In this report, we have excluded patients with other forms of immune-mediated ataxias such as paraneoplastic cerebellar degeneration and gluten ataxia where the treatment is different.

All patients were provided with patient information leaflet on PACA (produced for Sheffield Ataxia Centre, Sheffield Teaching Hospitals NHS Trust) and a leaflet about mycophenolate (produced by Arthritis Research UK). Mycophenolate was introduced at 500 mg twice daily and if tolerated and with normal safety blood monitoring (done every 2 weeks), the dose was increased to 1 g twice daily at 1 month. Patients were re-assessed on a 4 monthly basis with repeat safety blood monitoring done every 4 months thereafter.

### MR Spectroscopy

In addition to volumetric 3 T MR imaging, all patients underwent single-voxel H^1^ MR spectroscopy (MRS) of the cerebellum. This imaging protocol is in clinical use as part of the investigation of all patients with cerebellar ataxia attending the Sheffield Ataxia Centre. The brain imaging protocol for structural, volumetric and spectroscopy studies has been previously described [4]

Patients underwent baseline MRI including MRS of the vermis measuring *N*-acetyl-aspartate to creatine (NAA/Cr) area ratio, reflecting metabolic activity. Repeat MRS was performed after at least a year of treatment with mycophenolate. In common with other immune-mediated ataxias, the cerebellar vermis is primarily involved in PACA, and therefore, MRS results reported here are measurements from the cerebellar vermis [4, 5].

### Correlation of MRS with the Scale for the Assessment and Rating of Ataxia (SARA)

Forty consecutive patients with different types of ataxias, including genetic, immune and degenerative (cerebellar variant of multiple system atrophy), were included in the analysis for the purpose of this correlation. All of these patients had undergone MRS and SARA score as part of their clinical assessment whilst attending their follow-up appointments at the Sheffield Ataxia Centre.

### Statistical Analysis

Change in mean values between the groups was compared with Student’s two-tailed *t* test for unpaired samples. A value of *p* < 0.05 was considered significant. For the correlation analysis, Spearman’s rho was used as SARA is an ordinal measure.

## Results

### Demographics

The clinical characteristics of the 30 patients with PACA are summarised in Table 1. A total of 30 consecutive patients with PACA were included in the analysis. Of these, 22 patients started treatment with mycophenolate between January 2015 and the time of this report. Out of these 22 patients, 4 already had 2 MRS scans at 2 time points prior to starting the treatment, so their pre-treatment data was used as part of the control group’s MRS data which thus comprised 12 patients in total (the 4 that were subsequently treated plus 8 patients that are still on no treatment). The reasons for no treatment in the control group included delayed diagnosis of PACA and reluctance by the patient and family for the use of immunosuppressive medication. Some patients did not feel that their ataxia was bad enough to warrant treatment with mycophenolate. This was also reflected by the fact that the control group had higher NAA/Cr vermian baseline spectroscopy when compared to the treatment group.

The mean age of the 2 groups was 64.3 ± 14.4 years (treated) vs 63.3 ± 20.6 (untreated). The mean time difference between the baseline and the second MRS scan was 24 months in the treated group and 15.8 months in the untreated group. The mean duration of the ataxia in the treated group was 6.73 years (range 2–20) and in the untreated group 9.42 years (range 2–20). None of the above differences were significant.

None of the patients in the treatment group had to stop mycophenolate because of tolerability issues or side effects. Treatment was well tolerated; the only adverse effects reported were mild nausea (2 patients) after starting the medication. This subsided within a few weeks or with reduction of the dose.

### Correlation of MRS with the SARA

**Fig. 1:**
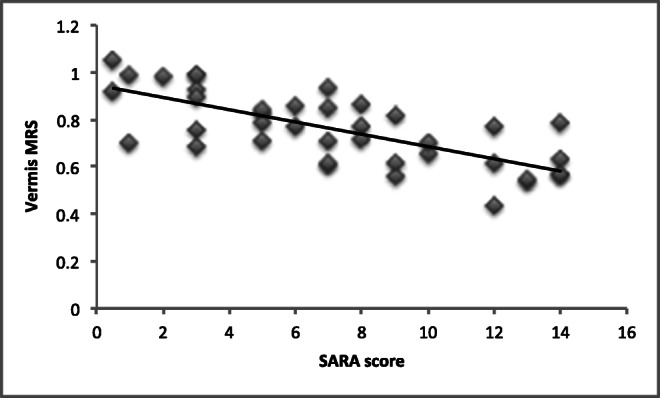
Graph depicting significant negative correlation between the sum of gait and stance scores from SARA (the higher the score the more severe the ataxia) vs NAA/Cr area ratio from MRS of the vermis (the higher the measurement, the better cerebellar functioning) in 40 patients with various types of ataxias. The correlation was highly significant with correlation coefficient rs = − 0.07, *p* = 0.00000129

There was a significant negative correlation using Spearman’s rho between the sum of gait and stance scores (a reflection of vermian functioning) from SARA Vs NAA/Cr area ratios from MRS of the vermis. The correlation was highly significant with the correlation coefficient rs = − 0.07, *p* = 0.00000129 (Fig. 1).

### MRS Results

**Fig. 2:**
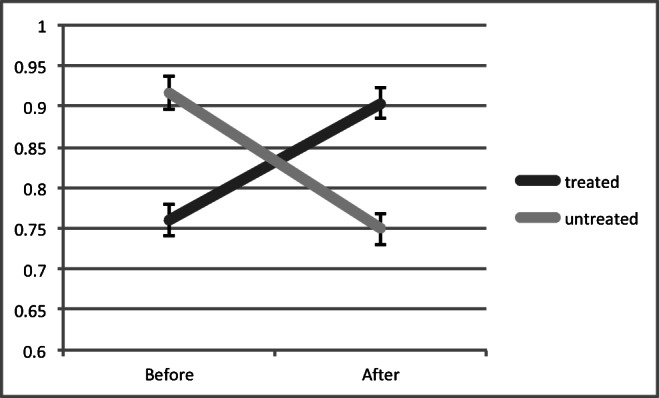
Change in MRS (NAA/Cr area ratio, vertical axis) after treatment (treated group) and in the untreated group. The difference was significant *p* < 0.0001 by Student’s *t* test

**Fig. 3:**
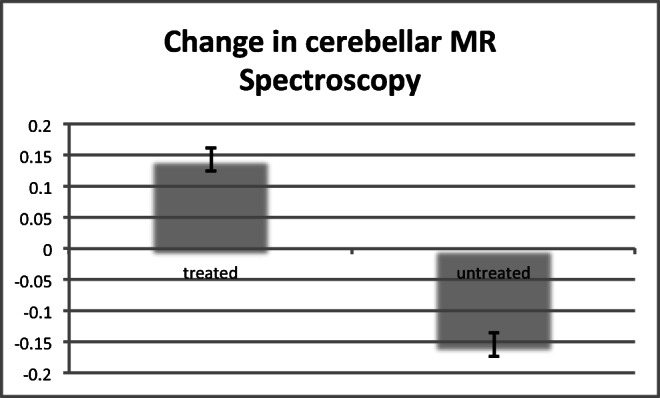
Change in MRS (NAA/Cr area ratio) from baseline in the treated and untreated groups

**Fig. 4:**
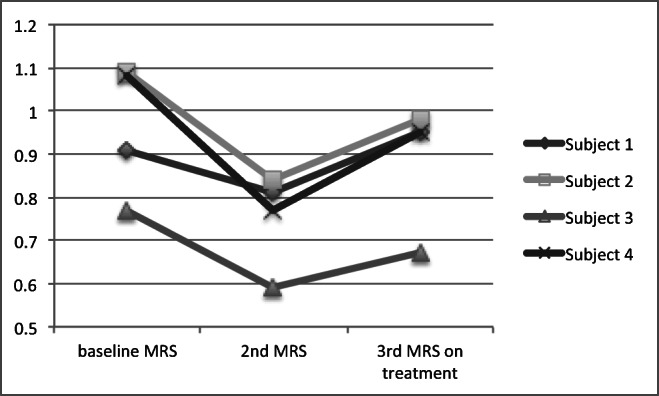
Change in MRS (NAA/Cr area ratio) in the 4 patients that were both in the control (baseline and 2nd MRS) and treatment groups (2nd and 3rd MRS), showing deteriorating NAA/Cr prior to treatment and improvement after treatment with mycophenolate

The mean change of the MRS within the vermis (NAA/Cr area ratio) in the treatment group was + 0.144 ± 0.09 and in the untreated group − 0.155 ± 0.06. The difference was significant (*p* < 0.0001 by Student’s *t* test). Fig. 2 illustrates these changes. Fig. 3 demonstrates the change from baseline in the 2 groups. In all 22 patients treated, the MRS within the vermis improved whereas in all of 12 patients untreated, the MRS worsened. For the 4 patients for which MRS data were available before and after treatment, Fig. 4 illustrates these changes. The MRS changes were associated with clinical improvement, and all the patients in the treatment group have continued treatment with mycophenolate.

## Discussion

We have demonstrated for the first time that treatment with mycophenolate in patients with suspected PACA results in increased NAA/Cr area ratio of the cerebellar vermis as measured with MRS. Such increase is associated with clinical improvement. We have demonstrated this by showing that NAA/Cr area ratio from the vermis inversely correlates with the SARA sub-score for gait and stance. This improvement with the use of mycophenolate supports the notion of an autoimmune aetiology for PACA and also that the ataxia is partially reversible with immunosuppression.

We selected mycophenolate for treating PACA as this drug has been in clinical use for many years, initially for the prophylaxis of acute rejection after organ transplantation and later to treat autoimmune diseases such as lupus nephritis and systemic vasculitis [6]. Over the last 15 years, we have gained experience with the use of this drug in patients with immune-mediated neurological diseases including neurological vasculitis, myasthenia gravis, neurosarcoidosis, inflammatory neuropathies, autoimmune encephalitis and neurological manifestations of gluten-related diseases in the context of refractory coeliac disease. Mycophenolate is well tolerated and serious side effects are extremely rare. It can be titrated to the effective dose (2 g per day) quickly, and its cost has been substantially reduced since generic preparations have become available.

Our experience in investigating and monitoring over 2000 patients with progressive ataxias over the last 25 years helped in the recognition of a cohort of patients suspected of having PACA [1, 7].

The term PACA is restricted to those ataxias where an antibody, if present, is not directly involved in the pathogenesis of the ataxia, neither does it act as a marker of another well-characterised immune ataxia (e.g. anti-Yo in the context of paraneoplastic ataxia). Such antibodies may not be neuronal but simply reflect additional autoimmune tendency (e.g. thyroid antibodies, intrinsic factor). Recently, diagnostic criteria for PACA have been published by an International Task Force on Immune-Mediated Cerebellar Ataxias [3].

We can confirm that the patients reported here would fulfil the diagnostic criteria for PACA as outlined in the Task Force publication.

Retrospective studies looking at the treatment of immune ataxias have utilized various immunomodulatory approaches including intravenous immunoglobulins, steroids, plasma exchange and rituximab [8]. One such retrospective study included 118 patients with suspected immune-mediated ataxias, 55 of which had non-paraneoplastic ataxia [9]. All patients had received some form of immunotherapy, and neurological improvement was reported in 54 patients. Regression analysis revealed that improvements were significantly more common amongst patients with non-paraneoplastic ataxias. There are no randomised control studies demonstrating the effectiveness of any of the above immunomodulatory approaches.

At the Sheffield Ataxia Centre, we prefer to avoid the use of long-term steroids in the context of PACA because of the potential long-term adverse effects. Short course of high dose steroids, used by some, is unlikely to demonstrate any immediate benefit and such an approach may create an erroneous impression that the disease is not responsive to immunomodulation. However, a subgroup of these patients may follow a very aggressive and rapidly progressive course. In this situation, steroids and intravenous immunoglobulins may be used at presentation before mycophenolate has its full effect. Such successful combinations of immunomodulation that include mycophenolate have already been published as case reports or small series [10–12].

We and others have used MRS as a useful biological marker of disease progression and disease response to therapeutic interventions [3, 4, 13–16]. In the current study, we also showed a correlation between MRS and SARA score. The advantage of MRS as a monitoring tool is that it can be easily performed as part of routine MR imaging, it is reproducible and relies on objective measurements such as the NAA/Cr area ratio. It therefore overcomes the disadvantages of the clinical scales (interrater variability, fluctuation of ataxia symptoms and signs due to fatigue, insensitive scales in severely disabled patients and ceiling effect).

MRS of the vermis in particular is more reliable and reproducible than that of the hemisphere as the lateral borders of the vermis guide the positioning of the voxel. As the vermis is primarily affected in immune ataxias including PACA, we recommend NAA/Cr area ratio from the vermis as the main monitoring tool. Whilst volumetric analysis may provide an accurate measure of cerebellar atrophy, atrophy may be considered as an end point of accumulated cerebellar insult, whereas MRS is sensitive to cerebellar injury and potential for recovery (cerebellar reserve).

There are limitations to this report. The results are observational and based on our experience in treating patients with suspected PACA. To our knowledge, no randomized control trial on the treatment of PACA has ever been performed and as ataxia is considered a rare disease such a study may be difficult to perform in a single centre. Furthermore, the availability of MRS of the cerebellum is very limited in the UK.

In conclusion, we have demonstrated for the first time the effectiveness of mycophenolate in increasing NAA/Cr area ratio of the cerebellar vermis as measured with MRS in patients with PACA. This increase correlates with clinical improvement. We have also shown that NAA/Cr area ratio correlates with a clinical scale of measuring ataxia (SARA). The results suggest that immune-mediated ataxias are potentially treatable and that there is a need for early diagnosis to prevent permanent neurological deficit and preserve cerebellar reserve. The recent publication of diagnostic criteria for PACA will hopefully aid neurologists in considering this diagnosis and treat with immunosuppression.
